# Supplementary material for: A UMLS-based spell checker for natural language processing in vaccine safety
Source: BMC Med Inform Decis Mak. 2007 Feb 12;7:3. doi: 10.1186/1472-6947-7-3 (PMC1805499; doi:10.1186/1472-6947-7-3)
Supplement: Additional file 13 — RAP application directory. Sets up directory for RAP (RDF application for PHP) source code [file 1472-6947-7-3-S13.gz › rap/doc/phpdoc/model/Literal.html]

xml version="1.0" encoding="iso-8859-1"?


Docs For Class Literal


## Class Literal

Description

Description |
Descendents
| Methods (details)

An RDF literal.

The literal supports the xml:lang and rdf:datatype property. For XML datatypes see: http://www.w3.org/TR/xmlschema-2/

History:

- access: public
- author: Chris Bizer <chris@bizer.de>
- author: Daniel Westphal <dawe@gmx.de>
- version: V0.9.1

Located in /model/Literal.php (line 26)

```
Object
   |
   --Node
      |
      --Literal
```

Direct descendents

Description |
Descendents
| Methods (details)

| Class | Description |
| --- | --- |
| ResLiteral | An RDF literal. |

Method Summary

Description |
Descendents |
Methods (details)

Literal
Literal
(string $str, [string $language = NULL])

boolean
equals
(object literal $that)

string
getDatatype
()

string
getLabel
()

string
getLanguage
()

void
setDatatype
(string $datatype)

void
setLanguage
(string $lang)

string
toString
()

Methods

Description |
Descendents |
Methods (details)

Constructor Literal (line 56)

Constructor

Literal

Literal
(string $str, [string $language = NULL])

- string
  $str: label of the literal
- string
  $language: optional language identifier

equals (line 139)

Checks if ihe literal equals another literal.

Two literals are equal, if they have the same label and they have the same language/datatype or both have no language/datatype property set.

- access: public

boolean

equals
(object literal $that)

- object literal
  $that

getDatatype (line 108)

Returns the datatype of the literal.

- return: datatype of the literal
- access: public

string

getDatatype
()

getLabel (line 75)

Returns the string value of the literal.

- return: value of the literal
- access: public

string

getLabel
()

getLanguage (line 86)

Returns the language of the literal.

- return: language of the literal
- access: public

string

getLanguage
()

setDatatype (line 122)

Sets the datatype of the literal.

Instead of datatype URI, you can also use an datatype shortcuts like STRING or INTEGER. The array $short\_datatype with the possible shortcuts is definded in ../constants.php

- access: public

void

setDatatype
(string $datatype)

- string
  $datatype: URI of XML datatype or datatype shortcut

setLanguage (line 97)

Sets the language of the literal.

- access: public

void

setLanguage
(string $lang)

- string
  $lang

toString (line 164)

Dumps literal.

- access: public

string

toString
()

---

Redefinition of:

Object::toString()
:   Serializes a object into a string

#### Inherited Methods


Inherited From Object

> Object::toString()

Documentation generated on Thu, 7 Jul 2005 13:41:52 +0200 by phpDocumentor 1.3.0RC3
